# Spatiotemporal Analysis of Food Production–Demand Mismatch in China and Implications for Agricultural Structural Adjustment

**DOI:** 10.3390/foods13131990

**Published:** 2024-06-24

**Authors:** Mengyao Long, Xiaoyu Xiong, Huilong Lin

**Affiliations:** State Key Laboratory of Herbage Improvement and Grassland Agro-Ecosystems, College of Pastoral Agriculture Science and Technology, Lanzhou University, Lanzhou 730020, China; 220220901870@lzu.edu.cn (M.L.); xiongxy19@lzu.edu.cn (X.X.)

**Keywords:** food equivalent unit (FEU), production-to-demand mismatch, grassland agriculture, spatial mismatch index, net primary productivity

## Abstract

Based on the food equivalent unit (FEU), this article analyzed Chinese food consumption patterns, spatial mismatch, and production potential to explore agricultural reform strategies. Assessing production–demand mismatch involved the spatial mismatch model, drawing data from statistical yearbooks. Calculations of food production potential utilized the CASA model and the Thornthwaite Memorial model, with net primary productivity (NPP) derived from remote sensing data as indicators. The results showed that livestock product consumption is on the rise, and the spatial mismatch index for herbivorous livestock products was the largest, ranging from 22.81 to 98.12 in 2019. The mismatched degree distribution of rations and food-consuming livestock products showed a trend of increasing on both sides, with the Hu Huanyong line as the center line. Production factors played a predominant role in food production-to-demand mismatch. Climatic productivity and actual productivity decreased from the southeast to northwest in space in 2019, and human activities significantly impacted productivity. When grassland agriculture is pursued as the adjustment orientation, the production potential can reach up to 4540.76 × 10^7^ kg·FEU. Moreover, a grassland agriculture plan was devised, prioritizing its development in the developed southern regions.

## 1. Introduction

China’s food security garners widespread international attention. Currently, the sustainable development of agriculture in China is faced with the dilemma of insufficient land and water resources, low quality, unbalanced regional allocation, and unreasonable use of resources [1]. At the same time, residents’ meat consumption is growing, triggering large imports of feed products. In 2022, China’s soybean imports reached 91.08 million tons, corn imports were 20.62 million tons, and other feed product imports were 1.94 million tons [2]. Without improvements in feed production, by 2050, China may need to import corn and soybeans equivalent to 97% to 100% of the global trade volume [3], posing a severe challenge to the grain supply. The issue of food security has evolved into a supply problem concerning animal products and feed grains. China has long adhered to the “pig-grain” agricultural model, but its food supply capacity has been constrained by both natural and human factors [4,5], with a large gap occurring in feed grain supply [6].

The economically developed countries of Western Europe have chosen to reduce the excessive consumption of meat and other animal products. On the other hand, China must seek efficient food production and supply strategies due to resource and environmental pressures, large population size, and diverse needs. Adjusting production according to demand helps optimize resource allocation and economic growth. Relying on the flexibility of policy implementation can improve residents’ quality of life and maintain social stability. At the same time, we must promote balanced regional development. Therefore, it is time for China’s agricultural development model to change. Grassland agriculture is the mainstream model of modern agriculture, which saves feed grain and has ecological functions [7]. By coupling grass and livestock systems, the ability of grassland agriculture to withstand market and natural risks can be enhanced [8]. Research on grassland agriculture in China rarely considers the combination of natural grassland and marginal land. Marginal land can also produce beneficial crops [9] to increase ecosystem benefits, mainly through the development of grassland agriculture [10].

The balance of food supply and demand is one of the essential symbols of national food security, and it is also a hot issue. The research perspectives of grain supply and demand in China mainly focus on production [11,12,13], consumption [11], supply and demand patterns [14,15,16], influencing factors [12,14,16], and supply and demand trend prediction [11,17]. The research methods include spatial autocorrelation analysis [15], GIS [13], the barycentric analysis model [12,16], the grain supply and demand model [13], the spatial mismatch index [14], the evaluation indicator system for food security [18], and the construction of coupling coordination degree models. Some research results show that the mismatch between grain production and demand in China has intensified [14], the focus on grain production and consumption has shifted, and the main influencing factors include nature, politics, the economy, and society [16,19]. From the perspective of research objects, most studies focus on cereals, and there are few studies on integrated cereal and livestock products. From the standpoint of the methods adopted by most studies, spatial autocorrelation analysis cannot directly measure the spatial matching degree of production and consumption of research objects. In contrast, while the process of gravity modeling is relatively simple, it can only reveal the spatial dislocation phenomenon of two elements in a macro perspective and cannot explain the intensity and patterns of local dislocation. From the standpoint of research content, although much research involves the temporal and spatial changes of food production and demand, there are few studies that analyze the impact of production or demand changes on food production and demand. The spatial mismatch hypothesis was first proposed by Kain [20], who used it to describe the spatial mismatch between residential space and employment space. In order to quantify the degree of separation between employment and housing, Martin [21] proposed the spatial mismatch index to closely link factors together. In recent years, the spatial mismatch model has been increasingly used to analyze the relationship between cultivated land resources and food production [22]. Considering the diversity and heterogeneity within a region, the spatial mismatch model can dynamically reveal the impact of factor changes on the mismatch of various food types. Therefore, this paper adopted the spatial mismatch model to study the mismatch of grain supply and demand in various provinces. 

Uniform metrics enable a comparative and scientific analysis of the food residents consume. The food equivalent unit (FEU) proposed by Ren and Hou [23] incorporates calculation methods and conversion formulas for both plant-based and animal-based foods. Thus, this study adopted FEU as an indicator to conduct a comprehensive investigation into three categories of food: rations, herbivorous livestock products, and grain-consuming livestock products. We delved into the evolution of food consumption patterns, the issue of mismatched production demands, and the food production potential of grassland agriculture in China. The research results will contribute valuable insights into the exploratory adjustment of agricultural structures and provide conclusive findings and recommendations. 

## 2. Materials and Methods

### 2.1. Study Area

**Figure 1:**
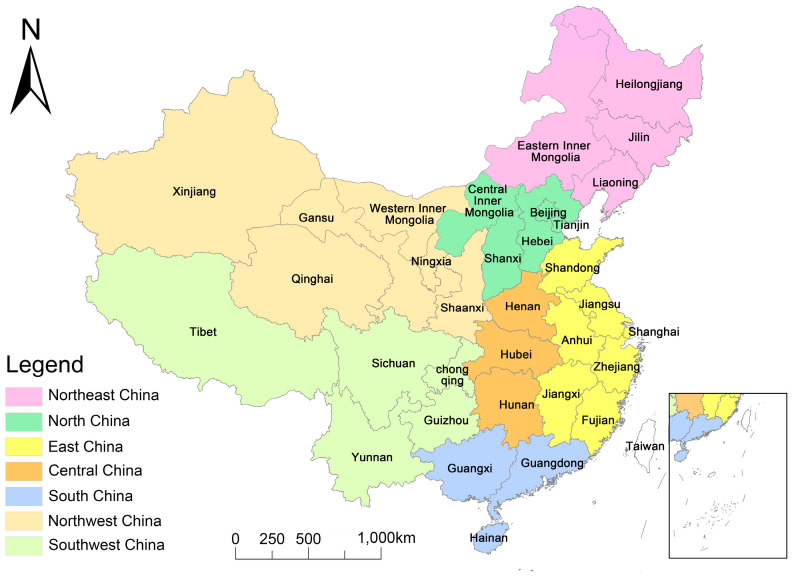
Thirty-one provincial-level administrative divisions of China, with 7 geographical divisions.

Thirty-one provincial-level administrative divisions of China (excluding Hong Kong, Macau, and Taiwan) were selected as the primary research unit, and the research was carried out in combination with seven administrative geographical divisions (see Figure 1).

### 2.2. Data Collection

Data on per capita food consumption in urban and rural areas and urban and rural populations nationwide and across 31 provincial-level administrative divisions, spanning from 1983 to 2020, were sourced from the China Statistical Yearbook and China Rural Statistical Yearbook. Additionally, food production and consumption data in 333 prefecture-level administrative districts (including prefecture-level cities, autonomous prefectures, unions, and regions) covering the period from 2007 to 2019 were obtained from corresponding provincial and prefectural-level city statistical yearbooks and national economic and social development statistical bulletins.

The research mainly used land use, photosynthetically active radiation, NDVI, and meteorological data. Data sources and processing are shown in Table 1.

### 2.3. Methodology

#### 2.3.1. Dynamics of Food Consumption Patterns from 1983 to 2030

Food types were categorized into rations, referring primarily to food grains for humans; grain-consuming livestock products, predominantly including pork, poultry, and poultry eggs; and herbivorous livestock products, mainly encompassing beef and mutton. Based on Ren and Hou’s research [23], food equivalents from 1983 to 2020 were calculated by multiplying plant and animal foods with their FEU coefficients. The grey prediction model, known for its strong data-processing ability and high accuracy [11], was used to forecast food consumption trends from 2021 to 2030.

#### 2.3.2. Spatiotemporal Analysis of Food Production-to-Demand Mismatch from 2007 to 2019

Beijing, Tianjin, Shanghai, and Chongqing were removed from the provincial-level administrative regions, and prefecture-level cities were taken as sub-regions. Based on the research results of Martin [21] and Han et al. [14], the spatial mismatch model was constructed as follows:

(1) $$S_{\mathrm{MI}}=100\times \frac{1}{p}\left|\sum_{i=1}^{n}\frac{c_{i}}{c}\times p-p_{i}\right|$$

where *S_MI_* represents the Spatial Mismatch Index, $c_{i}$ is the food production of the i sub-region, c is the food production in the provincial region, $p_{i}$ is the population of the i sub-region, and p is the population of the provincial region.

The hybrid *S_MI_*, including $M_{\mathrm{SMIC}}$ and $M_{\mathrm{SMIP}}$, was used to analyze the factors influencing mismatch. *S_MI_* after a change in production was calculated as follows:

(2) $$M_{\mathrm{SMIC}}=100\times \frac{1}{p_{m}}\left|\sum_{i=1}^{n}\frac{c_{i,\;m+1}}{c_{m+1}}\times p_{m}-p_{i,\;m}\right|$$

where $M_{\mathrm{SMIC}}$ represents *S_MI_* after changes in food production; $c_{i,\;m+1}$ denotes the food production of i sub-region in the m+1 year; $c_{m+1}$ signifies the food production of a provincial region in the m+1 year; $p_{i,\;m}$ represents the population of the i sub-region in the m year; and $p_{m}$ is the population of the provincial region in the m year.

Calculation of spatial mismatch index after demand changes was performed using the following equation:

(3) $$M_{\mathrm{SMIP}}=100\times \frac{1}{p_{m+1}}\left|\sum_{i=1}^{n}\frac{c_{i,\;m}}{c_{m}}\times p_{m+1}-p_{i,\;m+1}\right|$$

where $M_{\mathrm{SMIP}}$ represents the mismatch index after population changes; $p_{m+1}$ is the population of the provincial region in the m+1 year; $c_{i,\;m}$ denotes the food production of the i sub-region in the m year; $c_{m}$ signifies the food production of the provincial region in the m year; and $p_{i,\;m+1}$ represents the population of the i sub-region in the m+1 year.

We calculated $\Delta M_{\mathrm{SMIP}}$ and $\Delta M_{\mathrm{SMIC}}$ (the interannual rates of change for $M_{\mathrm{SMIP}}$ and $M_{\mathrm{SMIC}}$), with the difference between $\left|\Delta M_{\mathrm{SMIP}}\right|$ and $\left|\Delta M_{\mathrm{SMIC}}\right|$ denoted by ɳ. When ɳ>0, the dominant factor in spatial mismatch is demand; when ɳ<0, the dominant factor in spatial mismatch is production.

#### 2.3.3. The Computation of Food Production Potential

The main land use types were natural grasslands (intermediate and high-cover grassland) and marginal lands (such as bare rock, bare soil, and marshland). The Thornthwaite Memorial model and the CASA model were used to calculate climate productivity and actual productivity, respectively. The difference between them was expressed as Δ*NPP*, which represented the potential of the grassland [26].

Based on Lindeman’s law [27] and FEU [23], the potential total aboveground biomass was converted into food production potential (*PFP*). *PFP* was calculated as follows:

(4) $$\mathrm{PFP}=\frac{\Delta \mathrm{NPP}}{0.45}\times \frac{1}{\beta +1}\times 25\%\times \gamma$$

The conversion coefficient from plant biomass (g) to carbon (g C) is 0.45 [28]. The ratio of belowground biomass to aboveground biomass (β) is derived from the research results of Piao et al. [29]. The grassland utilization rate is 25% [30], and γ represents the food equivalent coefficient [31].

## 3. Results

### 3.1. Livestock Product Consumption Was Growing

**Figure 2:**
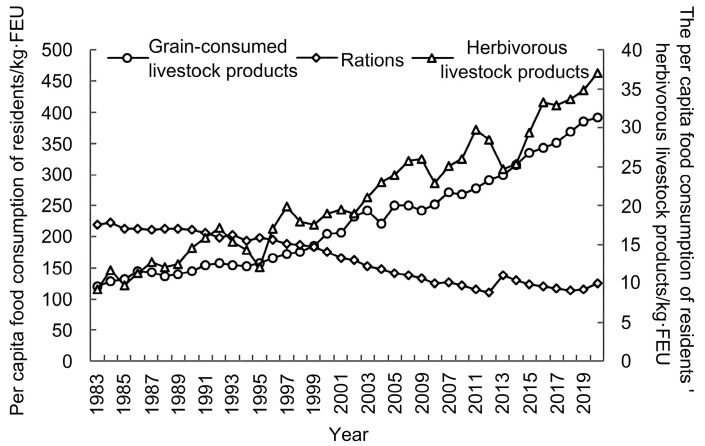
China’s per capita food consumption from 1983 to 2020.

From 1983 to 2020, the consumption of rations decreased from 219.21 kg·FEU to 124.52 kg·FEU, while that for grain-consuming livestock products increased from 119.91 kg·FEU to 391.63 kg·FEU and herbivorous livestock products increased from 9.27 kg·FEU to 37.06 kg·FEU. In 1997, Chinese residents’ meat consumption surpassed that of rations, reaching 77.49% of the total food consumption by 2020 (see Figure 2). By 2030, the per capita consumption of grain-consuming and herbivorous livestock products will reach 536.70 kg·FEU and 49.42 kg·FEU, respectively. Meanwhile, that for rations is anticipated to decline to 88.89 kg·FEU (see Supplementary Tables S1–S3). The predictive accuracy of the GM(1,1) model was good (see Supplementary Table S4). 

**Figure 3:**
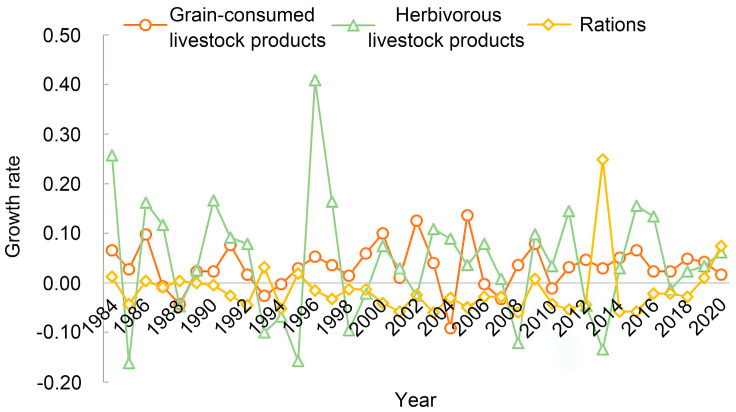
The annual growth rate of China’s per capita food consumption from 1983 to 2020.

The fluctuation in herbivorous livestock product consumption was most pronounced in 1997, with a growth rate of 16.51%. In contrast, the fluctuations in rations and grain-consuming livestock product consumption were relatively minor (see Figure 3).

### 3.2. A Serious Production–Demand Mismatch of Herbivorous Livestock Products

**Figure 4:**
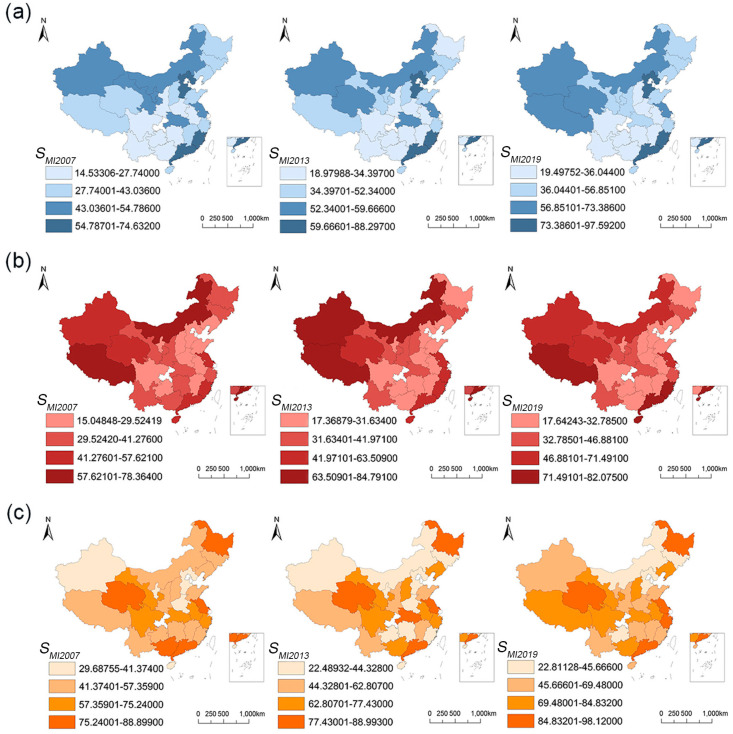
The distribution of *S_MI_* of rations (**a**), grain-consuming livestock products (**b**), and herbivorous livestock products (**c**) in each province.

From a temporal perspective, the *S_MI_* for the three food categories increased overall. Among them, the mismatch of herbivorous livestock products was the most serious, with *S_MI_* ranging from 22.81 to 98.12 in 2019 (see Figure 4). Provinces with severe ration mismatch in 2019 included Hebei, Fujian, and Guangdong, with *S_MI_* reaching 97.59, 90.77, and 90.12, respectively. The *S_MI_* of Tibet showed the most dramatic change in 2019, with a growth rate of 78.04% compared to 2007 (see Figure 4a). The spatial mismatch of grain-consuming livestock products became increasingly severe in most provinces. Provinces with a mean *S_MI_* for grain-consuming livestock products exceeding 60 included Inner Mongolia, Jiangsu, Guangdong, Hainan, Tibet, and Xinjiang, with Tibet having the highest average *S_MI_* of 81.74. In Hebei, Anhui, Hubei, Hunan, and Guangxi, the *S_MI_* of grain-consuming livestock products decreased during the fluctuation (see Figure 4b). The mean *S_MI_* for herbivorous livestock products exceeded 70 in nine provinces, with the mean *S_MI_* of Heilongjiang, Jiangsu, Hubei, Guangdong, and Qinghai exceeding 80. The herbivorous livestock product *S_MI_* of Inner Mongolia, Jilin, and Guizhou showed an apparent downward trend, and the *S_MI_* of herbivorous animal products in Tibet increased by more than 50% compared to 2007. Except for Guangxi Province, the spatial mismatch of herbivorous animal products increased gradually in most provinces of South China and East China (see Figure 4c).

Regarding spatial distribution, the degrees of mismatch for rations and grain-consuming livestock products showed a trend of increasing severity on both sides of the Hu Huanyong Line [32] (see Figure 4a,b), which connects Heihe City in northeastern China and Tengchong City in Southwest China and divides distinct natural environments [19,33]. In contrast, the spatial distribution of mismatch for herbivorous livestock products was more scattered (see Figure 4c).

**Figure 5:**
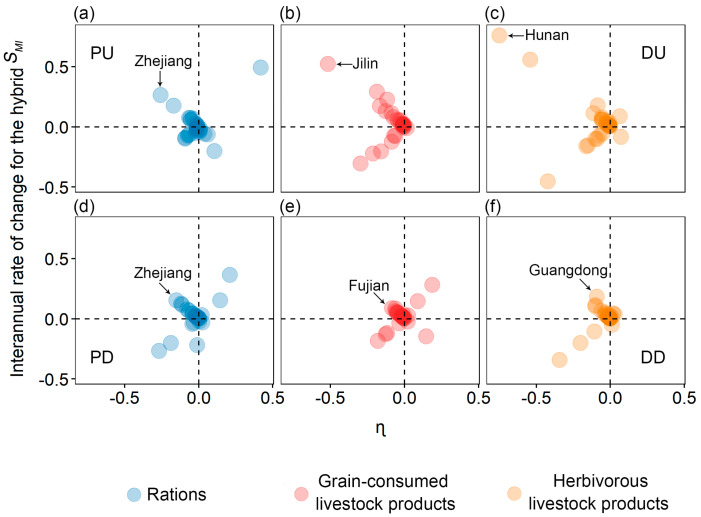
Influencing factors and changes in the food production-to-demand mismatch in 2007~2013 (**a**–**c**) and 2013~2019 (**d**–**f**) across 27 provinces. Explanation: Circles represent provinces. DU is the first quadrant in the four quadrants, indicating that the influencing factor is demand, and the hybrid *S_MI_* increases $\left|\Delta M_{\mathrm{SMIP}}\right|$; PU is the second quadrant, indicating that the influencing factor is production, and the hybrid *S_MI_* increases $\left|\Delta M_{\mathrm{SMIC}}\right|$; PD is the third quadrant, indicating that the influencing factor is production, and the hybrid *S_MI_* decreases $\left|\Delta M_{\mathrm{SMIC}}\right|$; DD is the fourth quadrant, indicating that the influencing factor is demand, and the hybrid *S_MI_* decreases $\left|\Delta M_{\mathrm{SMIP}}\right|$.

From 2007 to 2019, more than 62.96% of provinces experienced enormous impacts from production factors on the hybrid *S_MI_*, while the influence of demand factors remained relatively minor. Between 2007 and 2013, among the 27 provincial-level regions, 20 provinces were more influenced by production factors for rations (see Figure 5a), 26 for grain-consuming livestock products (see Figure 5b), and 25 for herbivorous livestock products (see Figure 5c). Additionally, 10 provinces were classified as PU type for rations, where production factors intensified spatial mismatch, 18 for grain-consuming livestock products, and 18 for herbivorous livestock products. Notably, Zhejiang experienced the highest increase in ration hybrid *S_MI_* (26.38%), Jilin had the highest increase in grain-consuming livestock product hybrid *S_MI_* (52.29%), and Hunan saw the highest increase in herbivorous livestock product hybrid *S_MI_* (75.95%). From 2013 to 2019, the number of provinces more influenced by production factors for rations (see Figure 5d), grain-consuming livestock products (see Figure 5e), and herbivorous livestock products (see Figure 5f) changed to 20, 22, and 23, respectively. Similarly, the number of PU-type provinces, where production factors intensified spatial mismatch, changed to 15, 18, and 20, respectively. Affected by production factors, Zhejiang had the highest increase in ration hybrid *S_MI_* (15.32%), Fujian had the highest increase in grain-consuming livestock product hybrid *S_MI_* (8.77%), and Guangdong had the highest increase in herbivorous livestock product hybrid *S_MI_* (18.26%).

### 3.3. Considerable Food Production Potential in Grasslands and Marginal Land

**Figure 6:**
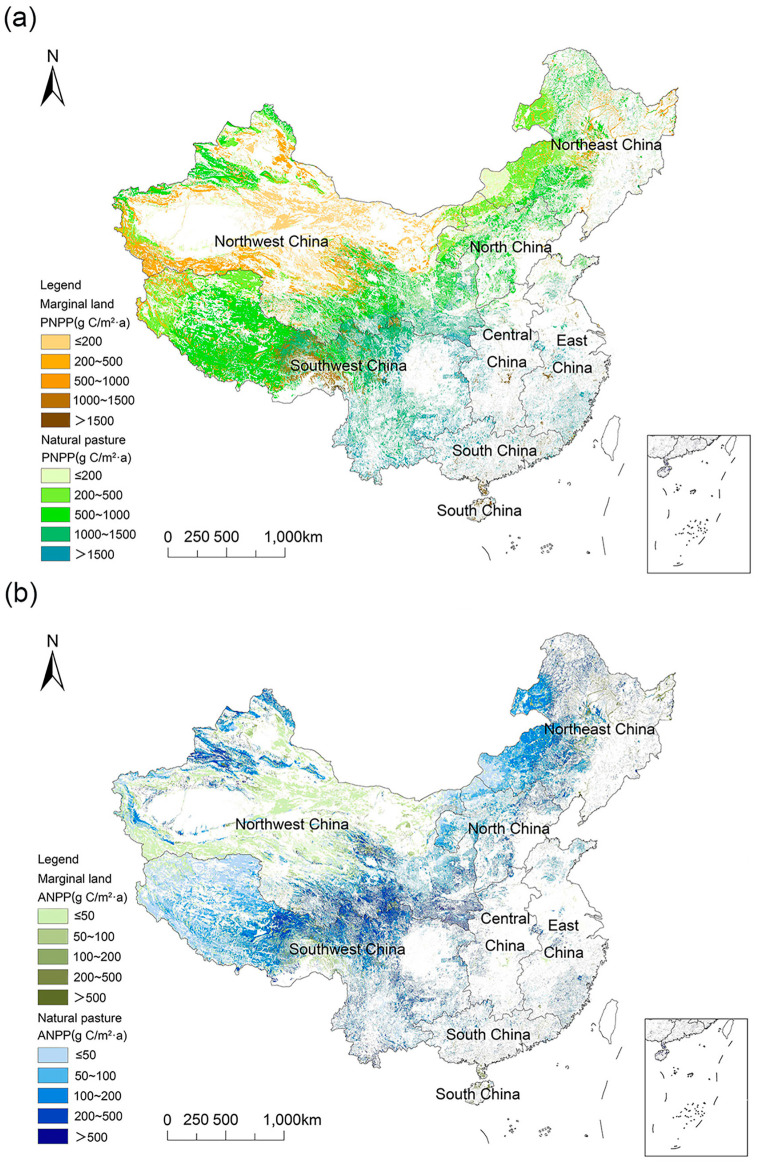
*PNPP* (**a**) and *ANPP* (**b**) were distributed in natural grassland and marginal land in 2019.

Due to the different hydrothermal conditions, resource endowments, and human activities in the north and the south, *PNPP* (see Figure 6a) and *ANPP* (see Figure 6b) in 2019 decreased from southeast to northwest. Human activities had a great influence on *NPP*.

Based on the total potential aboveground biomass in each region (see Supplementary Table S5), fully harnessing the production potential of natural grasslands and concurrently utilizing marginal land for grassland agriculture, the national food production potential can attain 4540.76 × 10^7^ kg·FEU. Among these, natural grasslands can produce 3777.98 × 10^7^ kg·FEU of food, representing 83.2% of the total. Regions such as Southwest, Northwest, Northeast, and North China showcase the substantial potential for food production, with the combined production capacity of these regions reaching 4385.24 × 10^7^ kg·FEU, accounting for 96.58% (see Table 2).

## 4. Discussion

### 4.1. Livestock Products Occupied an Essential Position in Food Consumption

From 1983 to 2020, per capita consumption of livestock products increased by about 3.32 times, while consumption of rations decreased by about 43.2%. By 2030, the demand for livestock products is predicted to be 6.59 times that of rations. Grain-consuming livestock products, mainly fed with grains in traditional agriculture, will account for 91.57% of total livestock product consumption (see Section 3.1). Although China’s population is aging, the proportion of significant income groups such as the young and middle-aged is still considerable, and animal products will continue to contribute a substantial proportion to the diet structure in the future. In addition, older people will have an increased demand for high-quality and safe meat. Therefore, the Chinese government needs to pay attention to improving the supply capacity of livestock products and the safety quality of livestock products based on the changes in demand. Due to limited cultivated land productivity and environmental constraints, the food supply is under tremendous pressure. There will be 230~260 million ton feed cereal gap by 2035 [6]. Although residents’ demand for rations is declining, the demand for feed grains in pig-grain agriculture would increase rigidly with an increase in demand for grain-consuming livestock products, and the sharp rise in food import demand would also significantly impact the domestic and international markets. Feed and grain supply are significantly challenged. Therefore, China’s traditional agricultural structure based on “pig grain” has been unable to adapt to the changing food consumption structure, and the farm structure needs to be adjusted urgently. The constraints of natural factors and the pressure on feed and grain supply mean that it is necessary to strengthen the innovation of efficient and environmentally friendly production technology and work toward the structural reform of the supply side to adapt to the demand side and the development of a variety of feed resources to improve the self-sufficiency rate and safety level of food. 

According to the Economic and Social Development Report of the National Bureau of Statistics of China, the per capita disposable income of Chinese residents in 2017, after deducting price factors, increased by 22.8 times over 1978 in real terms, with an average annual real growth of 8.5%. The increase in income level has gradually improved the diet structure of residents. It is worth noting that although the average annual consumption growth rate of herbivorous animal products from 1983 to 2020 (3.82%) exceeded the average annual consumption growth rate of grain-consuming animal products (3.25%), the overall annual growth rate fluctuated wildly. However, the proportion of herbivorous livestock products consumed per capita in 2020 was still only 8.64% (see Section 3.1). Compared with developed countries, the per capita consumption of cattle and mutton as part of China’s total meat consumption (including cattle, sheep, pig, and poultry) in 2020 accounted for 16.89%, which was lower than that of Australia (28.33%), Canada (26.57%), Japan (18.75%), the United Kingdom (24.72%), and the United States (26.18%). China’s per capita consumption of pork (51.13%) was higher than Australia (22.62%), Canada (22.71%), Japan (38.70%), the United Kingdom (25.69%), and the United States (23.62%). The per capita beef consumption of the United States was 6.24 times that of China, and that of Australia and Canada was 4.62 and 4.19 times that of China, respectively [34]. Compared with pork, beef and mutton played a less important role at the dining table of Chinese residents, and pork, which has a relatively high content of saturated fatty acids and cholesterol, should not be eaten too frequently. Hence, it must be reasonably mixed with different meats to maintain a healthy lifestyle. Beef and mutton, rich in high-quality protein, are substitutes for grain-consuming livestock products [35], with significant growth potential for consumption growth.

Furthermore, grass-fed beef contains healthier omega-3 fatty acids compared to grain-fed beef. Therefore, China should prioritize attention to herbivorous livestock products and formulate policies to support the production and marketing of herbivorous livestock products. Public health education, with a healthy diet as the goal, can guide residents toward moderate and diversified consumption, such as encouraging less excessive consumption of red meat and more significant consumption of grains and plant-based foods, encouraging the consumption pattern of residents toward the directions beneficial to the environment and health, and driving the supply gradually back to a reasonable range. There is consistency between claims to stimulate healthy eating patterns and to adapt production to meet demand in that they both contribute to the food system’s sustainability and public health.

### 4.2. Inspiration of Food Production-to-Demand Mismatch for Agricultural Structure Adjustment

Overall, the spatial mismatches of rations, grain-consuming livestock products, and herbivorous livestock products were on the rise, with herbivorous livestock products experiencing the most severe mismatch (see Section 3.2). Moving forward, greater attention should be directed towards herbivorous livestock products. The increasing food production-to-demand mismatch aligned with the advancement of the food consumption structure among Chinese residents, the migration of populations to developed areas, and the continuous rise of urbanization levels. 

The research underscored production as the primary factor contributing to the production-to-demand mismatch. Between 2007 and 2019, production factors significantly influenced the *S_MI_* of food in over 62.96% of provinces. After 2013, there was a reduction in the number of provinces where livestock products were more affected by production factors. Moreover, the rising degree of the hybrid *S_MI_* in PU-type provinces of the three types of food, where production factors contributed to increased mismatch, also declined (see Section 3.2). These trends may be attributed to the supply-side reform initiated in China at the end of 2012, suggesting that the reform may have somewhat mitigated the impact of production factors on spatial mismatch. Notably, although the number of provinces where rations were more affected by production factors remained unchanged after 2013, the number of PU-type provinces experiencing increasing mismatch due to production factors rose by 50% (see Section 3.2). Considering the stable arable land area, the rising output of rations, the decline in per capita ration consumption, and the implementation of the “selective two-child policy” in 2013, the supply-side reform was still deemed effective. 

The spatial distribution of mismatch degrees for rations and grain-consuming livestock products followed the Hu Huanyong Line, correlating with China’s regional natural geographic and socio-economic development differences. Conversely, the spatial distribution of production–demand mismatch for herbivorous livestock products was more dispersed, possibly due to regional dietary habits and cultural environments. If the spatial pattern of food production and demand continues to reflect the characteristics outlined by the Hu Huanyong Line, future challenges in alleviating spatial mismatch conflicts are anticipated. For example, “grain transport from the north to the south” was a typical production pattern of supply and demand contradiction, resulting in a severe waste of resources, unsustainable local environments [35], food loss, cost increases [36], and food safety issues [37]. Hence, addressing the spatial mismatch in food production and demand through supply-side reforms is imperative.

Residents’ demand for rations has declined, and the impact of supply-side reforms on rations has been more prominent. In contrast, both the market demand and the degree of mismatch for livestock products have increased. This indicates a need for further in-depth supply-side overhauls, especially for herbivorous livestock products. Grassland agriculture, a form of nutrient farming, exhibits strong environmental adaptability compared to traditional seed-based agriculture [8]. It utilizes the entire plant of crops and involves interactions between crops and livestock, leading to significant biodiversity [38]. Research has shown that the food production capacity of the warm-season rice/cold-season annual forage model in the southwestern region is more than three times higher than that of corresponding standard farmland [39]. Systematic coupling enhances the overall production level of grassland agriculture systems, balancing ecological and production concerns.

Considering the worsening supply situation of livestock products, the food production-to-demand mismatch (especially for herbivorous livestock products), and the abundant food production potential in the southern region, the development of grassland agriculture can play a crucial role. By alleviating the pressure on feed grain and ensuring the supply of herbivorous livestock products, grassland agriculture can address the gap in livestock product supply and improve the current state of production–demand mismatch. This approach points towards necessary adjustments in China’s agricultural structure for the future.

### 4.3. Grassland Agriculture Development Plan

**Figure 7:**
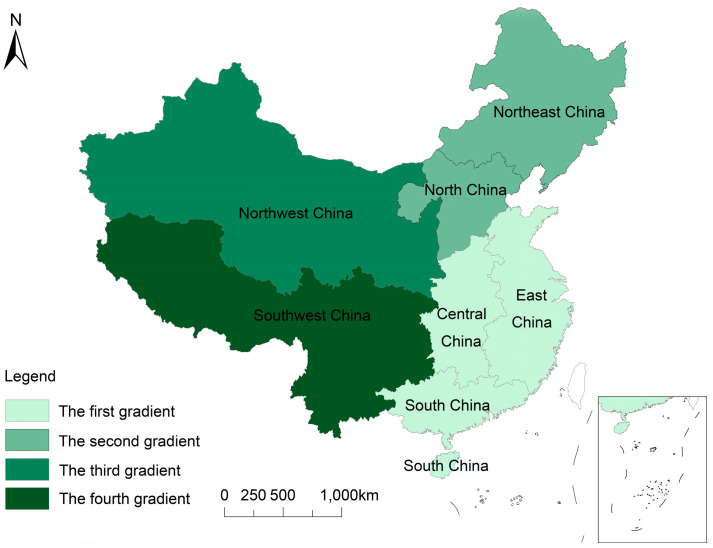
The gradients of developing grassland agriculture.

Using natural grassland and marginal land for grassland agriculture, the national food production potential reached 4540.76 × 10^7^ kg·FEU (see Section 3.3), of which natural grassland made a greater contribution. Four different grass-based agricultural development gradients were identified based on the food production potential of various regions (see Figure 7). At the same time, considering the regional resource endowment and production demand mismatch mode, we put forward the corresponding development strategy.

(1) The first gradient comprises central, southern, and eastern traditional agricultural regions. These areas have favorable water temperature conditions and great potential for grain production. Traditionally, food cultivation and animal husbandry are the main activities. However, due to the dense population and the shortage of land resources, the supply and demand for herbivorous animal products are seriously unbalanced. The development strategy is to comprehensively use natural grassland and marginal land and establish a differentiated animal husbandry model dominated by scale and efficiency. Local governments and agricultural departments should consider local needs and resource endowments, be responsible for policy formulation and implementation supervision, introduce high-quality forage varieties, promote crop rotation and intercropping, and improve production potential and soil fertility. Herders and farmers should use idle farmland and low-yield fields to rotate grassland, increase the proportion of herbivorous animals, and improve feed structure and animal husbandry efficiency. The new rural cooperative organization will expand production scale through land transfer, establish large-scale forage and feed production bases, and realize the integration of planting, production, processing, and sales. Transportation and market circulation enterprises, especially in provinces close to the Beijing–Tianjin, Yangtze River Delta, and Bohai Rim economic circles, should take advantage of convenient transportation and close production and marketing links to promote the circulation of forage and livestock products. Experts should provide technical support to help achieve efficient operation of large-scale forage feed production bases.

Source of funds: Government subsidies should be used to supervise the implementation and introduction of high-quality grass varieties. Agricultural development funds should promote efficient farming methods such as crop rotation and intercropping. Banks should provide loans to help herders and farmers develop herbivorous animal husbandry using idle arable land and low-yielding fields. Land transfer and large-scale production of new rural cooperative organizations should be promoted by attracting social investment.

(2) The second gradient: Northeast and North China. In these areas, grassland resources are abundant, grassland agriculture is developed, and the imbalance between the supply and demand of herbivorous livestock products is low. Livestock production mainly follows seasonal grazing patterns, supplemented by household rearing. Grassland is primarily distributed in agricultural and pastoral areas; reclamation is serious, soil erosion is intensified, and salinization and desertification occur in some areas. The development strategy of this gradient must strengthen grassland supervision and management to promote the upgrading and transformation of grassland animal husbandry. The northeast region should encourage the development of high-quality forage such as alfalfa and hay corn, relying on rich planting resources and reasonable livestock carrying capacity. Animal husbandry enterprises should focus on Holstein dairy cows and beef cattle, develop grass processing, straw modulation, and other industries, and promote silage technology and straw feed. Livestock enterprises in North China should rely on the advantages of Beijing–Tianjin–Hebei integration, leverage traditional production and processing advantages, and promote standardized large-scale breeding. The government and agricultural departments should reasonably plan the production capacity of pigs, cattle, and sheep, and farmers should optimize the breeding structure, develop herbivorous livestock such as cattle and sheep, and pursue diet-based animal husbandry development.

Source of funds: Government subsidies should be used for grassland supervision and management, revitalization of the alfalfa industry, and supporting silage corn and high-quality forage cultivation. Agricultural development funds should support livestock enterprises in developing supporting industries such as forage processing and straw modulation. Banks should provide loans to help livestock enterprises and herders optimize their breeding structure and develop herbivorous livestock such as cattle and sheep. Enterprise investment should be used to expand production scale and improve processing capacity.

(3) Northwest China is dominated by a temperate continental climate, fragile ecology, sparsely populated natural grassland, and marginal land area, with beef and mutton as the main meat consumed. Grassland has a good agricultural foundation and a large demand for herbivorous livestock products, but the production does not match the demand. The development strategy should focus on the development of safer and more sustainable grassland agriculture, rational use, and restoration of natural grasslands, grassy slopes, and marginal lands. Agricultural departments should guide the construction of water-saving artificial grassland and high-quality commodity alfalfa supply and grass seed industry bases. For degraded grassland, measures such as grazing prohibition, no-tillage replanting, fertilization, and controlled burning should be taken to promote restoration. Farmers and livestock enterprises should improve feed utilization through scientific formulation, quality feed supply, accurate feeding management, and regular monitoring. Local governments and agricultural departments should explore the transformation of ecological improvement in prohibited grazing areas into balanced grazing and livestock areas to promote the restoration of grassland agrarian production.

Source of funds: Government subsidies should be used to construct water-saving artificial grassland, high-quality commodity alfalfa supply bases, grass seed industry bases, and ecological improvement of natural grassland. Agricultural development funds should support grassland restoration measures such as grazing prohibition, no-tillage replanting, fertilization, and controlled burning. Banks should provide loans to help farmers and livestock enterprises use advanced equipment and technology to improve feed utilization and scientific management.

(4) Fourth gradient: southwest region. The southwest region has a subtropical monsoon climate and abundant water resources. It has the greatest potential for agricultural food production in grassland. The mismatch between the supply and demand of herbivorous animal products is relatively low, but there are serious mismatch problems in Sichuan and Tibet. Strengthening the development of the forage industry is an important measure to alleviate the mismatch of herbivorous livestock products. The development strategy should take both ecological protection and forage industry development as strategic objectives. It is suggested that under the premise of ensuring the stability of the cultivated land area, farmers should be allowed to use idle land to develop the forage industry. Local governments and agricultural departments should attach importance to ecological protection and implement projects such as returning grazing land to grassland, controlling the karst stony desertification of grassland, and returning farmland to forest and grassland. The production of herbivorous livestock and poultry and the planting division of forage varieties should be considered when delimiting prohibited breeding areas, restricted breeding areas, and protected areas. Livestock enterprises and large farms should improve feed utilization efficiency by improving feeding techniques and innovating feed formulations.

Source of funds: Government subsidies should be used to support ecological protection projects such as returning grazing land to grassland and comprehensively improving the karst rocky desertification of grasslands. Agricultural development funds should be used for scientific management and technical support to develop the forage industry and produce herbivorous livestock products. Banks should provide loans to help livestock enterprises and large farmers improve feeding technology and innovate feed formulations to improve feed utilization efficiency. Enterprise investment should be used to expand the scale of forage feed production and improve product quality.

There are some limitations in this study. First, the timeliness of the data: using data from five years ago may not fully reflect the current reality. Secondly, due to the absolute value of the model in this study, it is difficult to analyze the correlation and proportion of the mismatch between production and demand in detail, making it flawed to use the food space mismatch model for sensitivity analysis. Finally, the impact of climate change on grassland productivity has not been fully considered. For example, rising temperatures and changes in precipitation patterns may significantly alter grassland productivity in regions such as the northeast and northwest. The increasing frequency of extreme weather events may also cause damage to grasslands, and differences in climatic conditions across regions may lead to significant differences in the feasibility of grass-based agriculture. 

## 5. Conclusions

In summary, the research showed that the consumption of animal products in China is on the rise, with significant spatial mismatches, especially for herbivorous animal products. Production factors were the main causes of these mismatches. Grassland agriculture, particularly in the south, could alleviate these problems. Future studies should further explore the effects of climate change on grassland productivity and different grassland development patterns. Additionally, real-time production and consumption data could be obtained through blockchain technology to improve the timeliness of food spatial mismatch research.

## Figures and Tables

**Table 1:** Source and processing of primary remote sensing data and meteorological data.

Type	Source	Temporal Resolution	Spatial Resolution	Preprocess
Land Use Data	National Land Use Remote Sensing Monitoring Spatial Distribution Data (link)	Year	1 km	Processed using MODIS Reprojection Tools method
Photosynthetically Active Radiation Data	Environment Ecology Laboratory, BESS Model [24]	4 days	5 km	Processed using MODIS Reprojection Tools method, synthesized to annual, 1 km resolution with ArcGIS
NDVI Data	NASA, MODIS product (MOD13Q1) (link)	16 days	500 m	Processed using MODIS Reprojection Tools method, synthesized to annual, 1 km resolution with ArcGIS
Meteorological Data	National Meteorological Science Data Sharing Service Platform (link)	Year	-	Interpolated to 1 km resolution using ANUSPLIN (4.3) software [25]

**Table 2:** Food production potential in each region.

Region	Potential of Natural Grassland (×10^7^ kg·FEU)	Potential of Marginal Land (×10^7^ kg·FEU)
North China	203.29	16.95
Northeast China	453.17	99.86
East China	35.34	4.68
Central China	47.75	6.56
South China	48.68	12.51
Southwest China	2101.21	316.99
Northwest China	888.54	305.23

## Data Availability

The original contributions presented in the study are included in the article/Supplementary Material, further inquiries can be directed to the corresponding author.

## Supplementary Materials

The following supporting information can be downloaded at: https://www.mdpi.com/article/10.3390/foods13131990/s1, Table S1: Forecast results of national per capita rations consumption; Table S2: Forecast results of national per capita grain-fed livestock products consumption; Table S3: Forecast results of national per capita grass-fed livestock products consumption; Table S4: Accuracy test of grey prediction model; Table S5: Total potential aboveground biomass by region.
