# Supplementary material for: Spatiotemporal Analysis of Food Production–Demand Mismatch in China and Implications for Agricultural Structural Adjustment
Source: Foods. 2024 Jun 24;13(13):1990. doi: 10.3390/foods13131990 (PMC11241770; doi:10.3390/foods13131990)
Supplement: Supplementary file 1 [file foods-13-01990-s001.zip › foods-3020006-supplementary.pdf]

Supplementary Table S1. Forecast results of national per capita rations consumption.

Year	Actual Consumption/ kg·FEU	Predicted Consumption/ kg·FEU	Residual Error	Relative Error
1983	219.21			
1984	222.18	232.80	-10.62	-0.0478
1985	212.94	228.00	-15.06	-0.0707
1986	213.75	223.30	-9.55	-0.0447
1987	211.89	218.60	-6.71	-0.0317
1988	212.75	214.10	-1.35	-0.0064
1989	212.89	209.70	3.19	0.0150
1990	211.94	205.30	6.64	0.0313
1991	206.83	201.10	5.73	0.0277
1992	197.73	196.90	0.83	0.0042
1993	204.02	192.80	11.22	0.0550
1994	193.90	188.80	5.10	0.0263
1995	197.64	184.90	12.74	0.0645
1996	194.70	181.10	13.60	0.0699
1997	188.59	177.40	11.19	0.0593
1998	186.42	173.70	12.72	0.0682
1999	183.97	170.10	13.87	0.0754
2000	176.48	166.60	9.88	0.0560
2001	166.50	163.10	3.40	0.0204
2002	162.71	159.70	3.01	0.0185
2003	153.04	156.40	-3.36	-0.0220
2004	148.63	153.20	-4.57	-0.0308
2005	141.43	150.00	-8.57	-0.0606
2006	137.62	146.90	-9.28	-0.0675
2007	133.76	143.90	-10.14	-0.0758
2008	125.89	140.90	-15.01	-0.1193
2009	127.10	138.00	-10.90	-0.0858
2010	121.81	135.10	-13.29	-0.1091
2011	115.27	132.30	-17.03	-0.1477
2012	110.39	129.60	-19.21	-0.1740
2013	137.99	126.90	11.09	0.0804
2014	130.29	124.20	6.09	0.0467
2015	122.94	121.70	1.24	0.0101
2016	120.37	119.20	1.17	0.0097
2017	117.77	116.70	1.07	0.0090
2018	114.60	114.30	0.30	0.0026
2019	115.85	111.90	3.95	0.0341
2020	124.52	109.60	14.92	0.1199
2021		107.31		
2022		105.09		
2023		102.91		

2024	100.78
2025	98.69
2026	96.65
2027	94.65
2028	92.69
2029	90.77
2030	88.89

Supplementary Table S2. Forecast results of national per capita grain-fed livestock products consumption.

Year	Actual Consumption/ kg·FEU	Predicted Consumption/ kg·FEU	Residual Error	Relative Error
1983	119.91			
1984	127.85	118.80	9.05	0.0708
1985	131.58	122.70	8.88	0.0675
1986	144.45	126.80	17.65	0.1222
1987	143.48	131.10	12.38	0.0863
1988	137.34	135.40	1.94	0.0142
1989	140.67	139.90	0.77	0.0055
1990	144.04	144.60	-0.56	-0.0039
1991	155.07	149.40	5.67	0.0366
1992	157.84	154.40	3.44	0.0218
1993	153.92	159.50	-5.58	-0.0362
1994	153.65	164.90	-11.25	-0.0732
1995	158.38	170.40	-12.02	-0.0759
1996	166.72	176.00	-9.28	-0.0556
1997	172.84	181.90	-9.06	-0.0524
1998	175.43	188.00	-12.57	-0.0716
1999	185.92	194.20	-8.28	-0.0445
2000	204.67	200.70	3.97	0.0194
2001	206.74	207.40	-0.66	-0.0032
2002	232.97	214.30	18.67	0.0802
2003	242.75	221.40	21.35	0.0879
2004	220.79	228.80	-8.01	-0.0363
2005	251.15	236.50	14.65	0.0583
2006	250.99	244.30	6.69	0.0267
2007	242.88	252.50	-9.62	-0.0396
2008	251.62	260.90	-9.28	-0.0369
2009	271.45	271.40	0.05	0.0002
2010	268.91	278.60	-9.69	-0.0361
2011	277.37	287.90	-10.53	-0.0380
2012	290.56	297.50	-6.94	-0.0239
2013	299.13	307.40	-8.27	-0.0277
2014	314.73	317.60	-2.87	-0.0091
2015	335.75	328.20	7.55	0.0225
2016	343.50	339.10	4.40	0.0128
2017	352.03	350.40	1.63	0.0046

2018	369.13	362.10	7.03	0.0190
2019	385.19	374.20	10.99	0.0285
2020	391.63	386.70	4.93	0.0126
2021		399.55		
2022		412.87		
2023		426.63		
2024		440.85		
2025		455.55		
2026		470.73		
2027		486.42		
2028		502.63		
2029		519.38		
2030		536.70		

Supplementary Table S3. Forecast results of national per capita grass-fed livestock products consumption.

Year	Actual Consumption/ kg·FEU	Predicted Consumption/ kg·FEU	Residual Error	Relative Error
1983	9.27			
1984	11.66	11.44	0.22	0.0192
1985	9.80	11.81	-2.01	-0.2057
1986	11.38	12.19	-0.81	-0.0711
1987	12.73	12.58	0.15	0.0115
1988	12.15	12.99	-0.84	-0.0691
1989	12.47	13.41	-0.94	-0.0755
1990	14.55	13.84	0.71	0.0487
1991	15.88	14.29	1.59	0.0999
1992	17.13	14.75	2.38	0.1387
1993	15.42	15.23	0.19	0.0123
1994	14.37	15.72	-1.35	-0.0941
1995	12.12	16.23	-4.11	-0.3387
1996	17.08	16.76	0.32	0.0187
1997	19.90	17.30	2.60	0.1307
1998	18.02	17.86	0.16	0.0088
1999	17.63	18.43	-0.80	-0.0453
2000	18.97	19.03	-0.06	-0.0033
2001	19.53	19.64	-0.11	-0.0055
2002	19.04	20.28	-1.24	-0.0653
2003	21.11	20.94	0.17	0.0079
2004	23.02	21.61	1.41	0.0613
2005	23.89	22.31	1.58	0.0660
2006	25.76	23.03	2.73	0.1058
2007	26.01	23.78	2.23	0.0859
2008	22.87	24.54	-1.67	-0.0731
2009	25.12	25.34	-0.22	-0.0088
2010	25.99	26.16	-0.17	-0.0066
2011	29.77	27.00	2.77	0.0929

2012	28.46	27.88	0.58	0.0205
2013	24.67	28.78	-4.11	-0.1665
2014	25.41	29.71	-4.30	-0.1694
2015	29.36	30.67	-1.31	-0.0447
2016	33.29	31.66	1.63	0.0491
2017	32.89	32.68	0.21	0.0064
2018	33.69	33.74	-0.05	-0.0014
2019	34.85	34.83	0.02	0.0007
2020	37.06	35.95	1.11	0.0298
2021		37.12		
2022		38.32		
2023		39.55		
2024		40.83		
2025		42.15		
2026		43.52		
2027		44.92		
2028		46.37		
2029		47.87		
2030		49.42		

Supplementary Table S4. Accuracy test of grey prediction model.

Predicted Food Types	Posterior Difference Ratio C Value	Small Error Probability P Value
Rations	0.2557	0.9949
Grain-consumed Livestock Products	0.1231	0.9964
Herbivorous Livestock Products	0.2305	0.9884

Supplementary Table S5. Total potential aboveground biomass by region.

Types of Land	Region	Total potential above-ground biomass($\times 10^5$t)
Natural Grassland	North China	116.17
	Northeast China	258.95
	East China	20.19
	Central China	27.29
	South China	27.82
	Southwest China	1200.69
	Northwest China	507.74
Marginal Land	North China	9.69
	Northeast China	57.06
	East China	2.67
	Central China	3.75
	South China	7.15
	Southwest China	181.14
	Northwest China	174.42
